# Nucleotide diversity of the *Chlamydomonas reinhardtii *plastid genome: addressing the mutational-hazard hypothesis

**DOI:** 10.1186/1471-2148-9-120

**Published:** 2009-05-27

**Authors:** David Roy Smith, Robert W Lee

**Affiliations:** 1 Department of Biology, Dalhousie University, Halifax, Nova Scotia, Canada

## Abstract

**Background:**

The mutational-hazard hypothesis argues that the noncoding-DNA content of a genome is a consequence of the mutation rate (μ) and the effective number of genes per locus in the population (*N*_*g*_). The hypothesis predicts that genomes with a high *N*_*g*_μ will be more compact than those with a small *N*_*g*_μ. Approximations of *N*_*g*_μ can be gained by measuring the nucleotide diversity at silent sites (π_silent_). We addressed the mutation-hazard hypothesis apropos plastid-genome evolution by measuring π_silent _of the *Chlamydomonas reinhardtii *plastid DNA (ptDNA), the most noncoding-DNA-dense plastid genome observed to date. The data presented here in conjunction with previously published values of π_silent _for the *C. reinhardtii *mitochondrial and nuclear genomes, which are respectively compact and bloated, allow for a complete analysis of nucleotide diversity and genome compactness in all three genetic compartments of this model organism.

**Results:**

In *C. reinhardtii*, the mean estimate of π_silent _forthe ptDNA (14.5 × 10^-3^) is less than that of the nuclear DNA (32 × 10^-3^) and greater than that of the mitochondrial DNA (8.5 × 10^-3^). On average, *C. reinhardtii *has ~4 times more silent-site ptDNA diversity than the mean value reported for land plants, which have more compact plastid genomes. The silent-site nucleotide diversity of the different ptDNA loci that were studied varied significantly: from 0 to 71 × 10^-3 ^for synonymous sites and from 0 to 42 × 10^-3 ^for intergenic regions.

**Conclusion:**

Our findings on silent-site ptDNA diversity are inconsistent with what would be expected under the mutational-hazard hypothesis and go against the documented trend in other systems of π_silent _positively correlating with genome compactness. Overall, we highlight the lack of reliable nucleotide-diversity measurements for ptDNA and hope that the values presented here will act as sound data for future research concerning the mutational-hazard hypothesis and plastid evolution in general.

## Background

The magnitude of noncoding DNA in genomes can differ dramatically both among and within evolutionary lineages. This statement holds true for prokaryotic genomes and for the nuclear, mitochondrial, and plastid genomes of eukaryotes. The mutational-hazard (or mutational-burden) hypothesis [1] asserts that much of this observed variation in genome compactness can be explained by the product of the effective genetic population size (represented in this study as the effective number of gene copies at a locus [*N*_*g*_], not individuals) and the mutation rate (μ). The hypothesis maintains that an allele with more noncoding nucleotides than an alternative allele will be selectively disadvantageous because the excess noncoding DNA can accumulate hazardous mutations that may negatively impact gene function; the burden (or selective disadvantage) of the allele containing the surplus of noncoding DNA is determined by μ and the number of additional noncoding nucleotides in the larger allele that can affect gene function. The hypothesis proposes that natural selection is more efficient at perceiving the burden of the expanded allele when *N*_*g *_is large; thus, genomes with a high *N*_*g*_μ are predicted to be more compact than those with a small *N*_*g*_μ.

Population-genetic theory tells us that at mutation-drift equilibrium the nucleotide diversity at neutral sites (π_neutral_) is equal to *2N*_*g *_μ (where *N*_*g *_of uniparentally inherited organelle genes is thought to be about half that of haploid nuclear genes [2]). Estimates of π_neutral _can be acquired by measuring the nucleotide diversity at silent sites (π_silent_), which include noncoding sites and the synonymous sites of protein-coding DNA. Because there are many factors that can cause *N*_*g *_to deviate from these neutral expectations, such as the influence of natural selection on linked variation, the only way to gain insight into 2*N*_*g*_μ is through empirical observation, i.e., by measuring π_silent_.

As predicted by the mutational-hazard hypothesis, studies have found a positive correlation between π_silent _and genome compactness: for the coding-rich DNA of prokaryotes π_silent _is generally > 50 × 10^-3^; for the more noncoding-dense nuclear DNA (nucDNA) of land plants π_silent _is in the range of 3 × 10^-3 ^to 15 × 10^-3^; and for the nuclear genomes of vertebrates, which abound with noncoding DNA, π_silent _tends to be ~3 × 10^-3 ^[3]. Similar trends are also observed for mitochondrial genomes: in the streamlined mitochondrial DNA (mtDNA) of mammals π_silent _is ~40 × 10^-3^, whereas that for land-plant mtDNA, which is predominantly noncoding, is predicted to be < 0.4 × 10^-3 ^[4]. The contrast in π_silent _between mammalian and land-plant mtDNA is thought to be a consequence of the high mutation rate in the former and the low mutation rate in the latter. Mutation rate has also been invoked to explain why, despite similar proposed values of *N*_*g*_, the mitochondrial and nuclear genomes of mammals have opposite coding densities – in mammals estimates of μ for mtDNA are roughly 30 times those for nucDNA [4].

It is speculated that π_silent_ for plastid DNA (ptDNA) also correlates positively with genome compactness [1,4]; however, this issue has not been formally addressed because there are very few ptDNA sequences for which both π_silent_ and genome-compactness data are available — we are aware of only two plastid genomes for which these two statistics are published: those of Arabidopsis thaliana and Cycas taitungensis; moreover, the silent-site diversities for these two genomes were derived in each case from only a single locus and, therefore, may have been unrepresentative because of a low sampling bias (see the supplementary material of Lynch et al. [4]).

Of the 146 complete plastid-genome sequences available at the National Center for Biotechnology Information (NCBI; [5]) as of November 2008, the noncoding-DNA content ranges from 5%, in the apicomplexan *Eimeria tenella*, to 56%, in the unicellular green alga *Chlamydomonas reinhardtii *– a complete compilation is shown in Supplementary Table S1 [see Additional file 1]. Intriguingly, four of the five most bloated ptDNA sequences come from the Chlorophyta (a phylum containing most of the green-algal diversification), suggesting that this lineage is ideal for evaluating the mutational-hazard hypothesis vis-à-vis ptDNA. However, no studies as of yet have measured silent-site ptDNA diversity from the Chlorophyta. *C. reinhardtii*, a unicellular haploid alga, is a good candidate for investigating ptDNA diversity because it has a large (204 kilobases [kb]) and expanded plastid genome, and it is also a model organism for studying plastids and their photosynthetic processes [6]. From the viewpoint of the mutational-hazard hypothesis, π_silent _in the *C. reinhardtii *ptDNA should be less than that of more compact organelle genomes.

A previous study on *C. reinhardtii *[7] measured nucleotide diversity in its mitochondrial and nuclear genomes, which are respectively streamlined (~16–20 kb and ~20–30% noncoding, depending on the presence of optional introns) and bloated (~121 Megabases and ~83% noncoding). The mutational-hazard hypothesis would have forecasted π_silent _for the mitochondrial genome to be greater than that of the nuclear genome, but instead π_silent _for the mtDNA was found to be 4 times smaller than that of the nucDNA (8.5 × 10^-3 ^vs. 32 × 10^-3^). Although these findings were in opposition to the mutational-hazard hypothesis, it was suggested that introns in the mtDNA impose a greater burden than those in the nuclear DNA and predicted that the same may be true for the mitochondrial intergenic regions [7].

It would be interesting to see for *C. reinhardtii *how values of π_silent _for the plastid genome compare to those of the mitochondrial and nuclear genomes. When considering the fraction of noncoding DNA in each of these genomes, the mutational-hazard hypothesis would predict π_silent _for the ptDNA to be smaller than that of the mtDNA and larger than that of the nucDNA. But it is already known, as discussed above, that this is not the case: in *C. reinhardtii *the mtDNA has less silent-site diversity than the nucDNA. If the noncoding regions in the plastid genome carry an inflated burden, as suggested for those in the mtDNA, then we would expect a very low value of π_silent _for the ptDNA, much smaller than that of the mtDNA (i.e., << 8.5 × 10^-3^). However, if π_silent _for the ptDNA is significantly larger than that of the mtDNA but still smaller than that of the nucDNA, it will be difficult to find any support in our data for the mutational-hazard hypothesis. In addition, silent-site ptDNA diversity data from *C. reinhardtii *will allow for a comparison of the π_silent _values for the three genetic compartments of this species with those of *Arabidopsis lyrata*, the only other species for which reliable π_silent _estimates from ptDNA, mtDNA, and nucDNA are published [8]. Thus, to directly confront these issues, we measured π_silent _from the ptDNA of various geographical isolates of *C. reinhardtii*.

## Results

### Strains and their genetic loci

**Figure 1:**
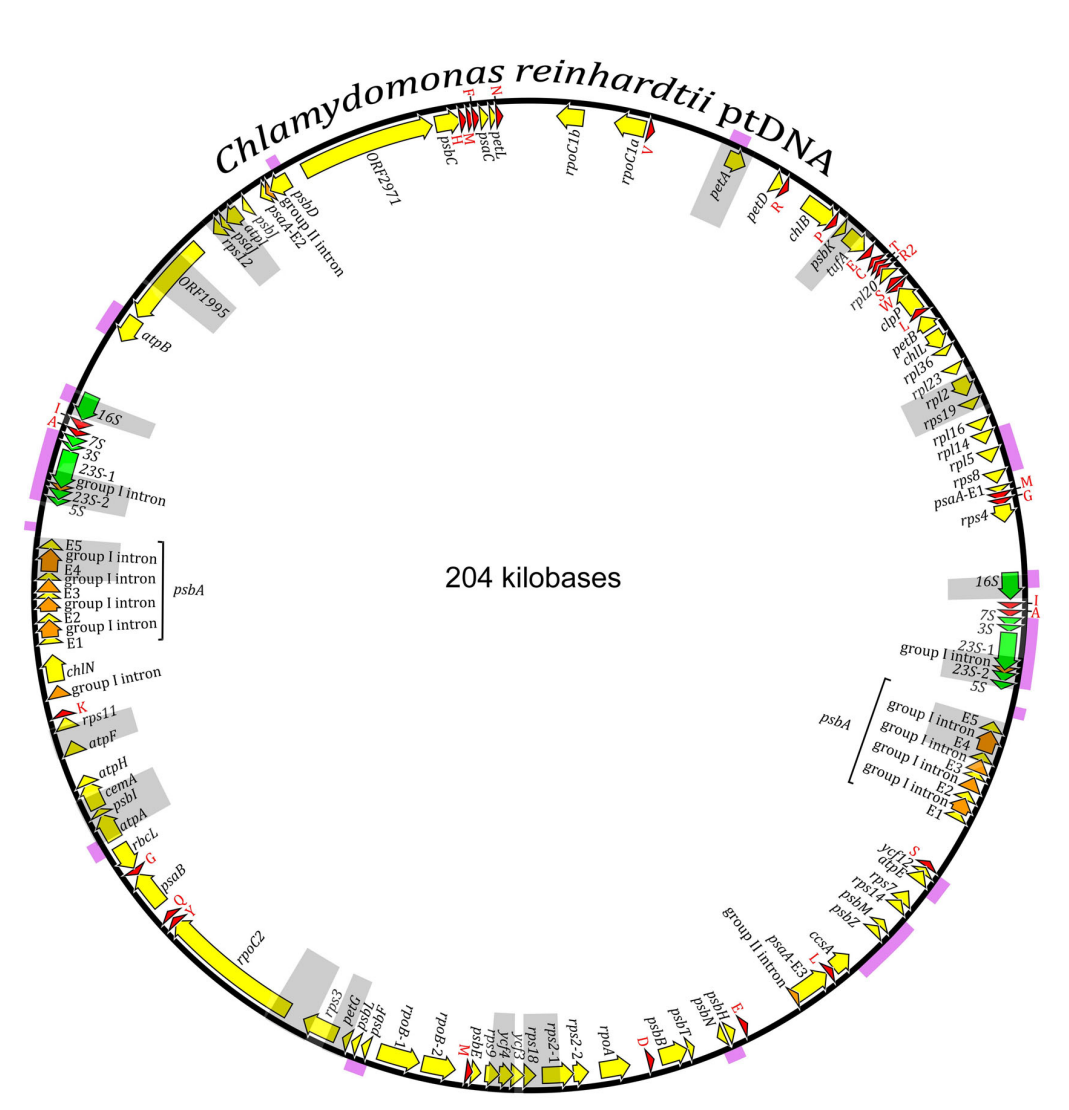
**Genetic map of the *Chlamydomonas reinhardtii *plastid genome**. Protein-coding regions are yellow and their exons are labeled with an "E" followed by a number denoting their position within the gene. Introns and their associated open reading frames are orange. Transfer RNA-coding regions are red and are represented by the single-letter code of the amino acid they specify. Ribosomal RNA-coding regions are green. All of the coding regions are shaped into arrows that denote their transcriptional polarities. Gray blocks correspond to the loci that were sequenced and used for measuring nucleotide diversity. The portions of the *C. reinhardtii *strain CC-2290 plastid genome that were data mined from GenBank are highlighted in pink.

For our analysis we employed seven geographical isolates of *C. reinhardtii*, which are listed in Table 1. These are the same isolates that were previously used for calculating π_silent _of the mtDNA and nucDNA. From each isolate, 14 distinct ptDNA regions were sequenced, amounting to 9.5 kb, 7.2 kb, and 2.7 kb of intergenic, protein-coding, and rRNA-coding ptDNA, respectively. A genetic map of the *C. reinhardtii *plastid genome highlighting these regions is shown in Figure 1.

**Table 1:** *Chlamydomonas reinhardtii *strains employed in this study.

**Strain**	**Mating Type**	**Strain Synonym**	**Geographical Origin (USA)**
CC-503	*mt*^+^	cw92	Amherst, Massachusetts
CC-1373	*mt*^+^	*C. smithii*	South Deerfield, Massachusetts
CC-1952	*mt*^-^	S1-C5	Plymouth, Minnesota
CC-2290^a^	*mt*^-^	S1-D2	Plymouth, Minnesota
CC-2342	*mt*^-^	Jarvik 6	Pittsburgh, Pennsylvania
CC-2344	*mt*^+^	Jarvik 356	Malvern, Pennsylvania
CC-2343	*mt*^+^	Jarvik 124	Melbourne, Florida
CC-2931	*mt*^-^	Harris 6	Durham, North Carolina

^a ^PtDNA sequences were data mined from this strain and compared only to those of CC-503

We also produced a complete plastid-genome sequence for *C. reinhardtii *strain CC-503 (one of the isolates described in Table 1) by assembling ptDNA trace files generated by the *C. reinhardtii *nuclear-genome sequencing project [9,10]. The earlier complete *C. reinhardtii *ptDNA sequence deposited at GenBank (accession# NC_005353) is a mosaic derived by linking the sequence data of various laboratory strains, most of which came from the "Ebersold-Levine" wild-type background of *C. reinhardtii *[11] – it is ideal to avoid using NC_005353 when calculating π_silent _because sequence differences have been found between the ptDNA of some laboratory strains [11]. A comparison of our CC-503 ptDNA sequence with NC_005353 reveals 471 single-nucleotide differences and 955 single-site indels; moreover, when the 14 ptDNA regions sequenced from the geographical isolates were sequenced from two additional laboratory strains belonging to the "Ebersold-Levine" wild-type background (CC-277 and CC-2454) the resulting data were identical to our CC-503-generated sequence but showed differences with NC_005353, suggesting that at least some of the discrepancies between CC-503 and NC_005353 are the result of sequencing errors in the latter. Thus, at present, the *C. reinhardtii *plastid-genome sequence presented here appears to the most accurate.

Twenty kilobases of intergenic ptDNA-sequence data from an additional geographical isolate of *C. reinhardtii *(CC-2290) was obtained by data mining plastid sequences from GenBank (Figure 1 and Supplementary Table S2 [see Additional file 2]); because very little of these sequence data overlap with the 14 regions described above they were only compared to the ptDNA of CC-503.

### Nucleotide diversity

Nucleotide-diversity measurements for the three genetic compartments of *C. reinhardtii *are summarized in Table 2. Net values of π_silent _for the plastid genome are 14.5 × 10^-3 ^when indels are removed from the alignment and 18.4 × 10^-3 ^when indels are included and counted as polymorphisms (π_silent+_); note, indels involving more than one nucleotide are considered to be a single polymorphic site. These values of π_silent _and π_silent+ _for the ptDNA are, respectively, 1.7 and 2 times those of the mtDNA, and 0.45 and 0.5 times those of the nucDNA. The nucleotide diversity values for the individual intergenic regions that were analyzed (outlined in Table 3) range from 0 to 41.6 × 10^-3 ^(average π_intergenic _= 11.3 × 10^-3^), and the π_intergenic+ _measurements for these same regions span from 0 to 53.2 × 10^-3 ^(average π_intergenic+ _= 14.4 × 10^-3^). The synonymous-site nucleotide diversity of the different protein-coding genes that were sequenced varies from 0 to 71.1 × 10^-3 ^(average π_syn _= 7.8 × 10^-3^; Table 3). Relative to the mitochondrial and nuclear genomes, the ptDNA shows more variance in nucleotide diversity among different regions: π_intergenic _and π_syn _of the various mtDNA loci range from 0 to 17.3 × 10^-3 ^(average = 11.4 × 10^-3^) and from 1.6 × 10^-3 ^to 15.3 × 10^-3 ^(average = 8.1 × 10^-3^), respectively; and for the nucDNA, π_intergenic _varies from 21.6 × 10^-3 ^to 58.3 × 10^-3 ^(average = 36.1 × 10^-3^) and π_syn _extends from 2.8 × 10^-3 ^to 41.1 × 10^-3 ^(average = 20.9 × 10^-3^). The ptDNA diversity of the rRNA-coding regions that were analyzed is 1.8 × 10^-3^, which is slightly lower than that of the mtDNA rRNA-coding regions (2.4 × 10^-3^) – at present there are no nucleotide diversity data for rRNA-coding nucDNA.

**Table 2:** Nucleotide diversity for the plastid, mitochondrial, and nuclear genomes of Chlamydomonas reinhardtii.

	**Protein-coding regions**			**Intronic/intergenic regions^e^**			**Silent sites^f^**		
	ptDNA	mtDNA	nucDNA	ptDNA	mtDNA	nucDNA	ptDNA	mtDNA	nucDNA
**# of sites^a^**	7272	8160	1623	9438	2457	4510	16710	5550	5051
***S***	45	44	26	276	58	355	321	104	377
**# of Indels^b^** **(length nt)**	1 (21)	1 (6)	1 (9)	85 (1672)	9 (23)	47 (216)	86 (1679)	11 (31)	48 (222)
**π^c ^× 10^-3^** **(SD × 10^-3^)**	2.82 (0.34)	2.06 (0.43)	6.02 (0.99)	15.17 (1.70)	8.92 (1.88)	33.50 (3.15)	14.53 (1.18)	8.51 (1.03)	32.29 (3.01)
**π_+_^d ^× 10^-3^** **(SD × 10^-3^)**	---	---	---	19.54 (2.03)	10.29 (2.02)	38.63 (3.70)	18.36 (1.40)	9.23 (1.96)	36.00 (3.51)
**π_syn _× 10^-3^**	8.46	8.52	19.57	---	---	---	---	---	---
**π_nsyn _× 10^-3^**	1.14	0	1.42	---	---	---	---	---	---

Note: *S*, number of segregating (i.e., polymorphic) sites; Indels, insertion-deletion events; π, nucleotide diversity; π_+_, nucleotide diversity including both polymorphic sites and insertion-deletion events; π_syn_, nucleotide diversity at synonymous sites; π_nsyn_, nucleotide diversity at nonsynonymous sites; SD, standard deviation. Mitochondrial- and nuclear-genome data come from [7].

^a ^Comprises all sites in the nucleotide alignment, including those with indels.

^b ^Indels involving more than 1 nucleotide are counted as a single event. Indel length includes the sum of all indels and includes consecutive indel events.

^c ^Only includes sites in the alignment without indels.

^d ^Considers all sites, including those with indels. Consecutive indels are counted as a single polymorphic event. Indel states were measured using a multiallelic approach.

^e ^For the ptDNA and mtDNA these values were generated from intergenic nucleotide sites, whereas for the nucDNA they came from intronic sites.

^f ^Includes noncoding and synonymous sites; π_+ _values for the mtDNA and nucDNA differ slightly from those previously reported [7] due to a different interpretation of indel sites in nucleotide-diversity calculations.

The silent-site ptDNA diversity between CC-2290 (the strain from which ptDNA sequences were data mined) and CC-503 is 6.5 × 10^-3 ^and π_silent+ _is 18.8 × 10^-3^; these values indicate that in the regions compared between CC-2290 and CC-503, single-site substitution differences are less frequent and indels are more frequent per site than in the regions compared in the group including CC-503 and the other six geographical isolates.

The various plastid-DNA loci were examined for traces of selection using Tajima's *D*-test (Table 3), which compares the average number of nucleotide differences between pairs of sequences (i.e., π) to the total number of segregating sites (*S*) [12]. Tajima's *D *is positive for the protein-coding genes *atpA*, *cemA*, *psbA*, *rpoC2*, and *rpl2 *and negative for *atpI*, *orf1995*, *rps9*, and *ycf3*. All of the analyzed intergenic regions show positive values for Tajima's *D*, with the exception of the *atpF*-*rps11 *intergenic spacer, which has a negative *D *value. The only cases where Tajima's *D*-test is statistically significant are for the protein-coding gene *rpoC2 *(Tajima's *D *= 2.03, P value < 0.05) and the region between the rRNA-coding genes *23S-1 *and *23-2 *(Tajima's *D *= 2.10, P value < 0.05).

**Table 3:** Nucleotide diversity (by region) in the *Chlamydomonas reinhardtii *plastid genome.

	**# of sites^a^**	***S***	**# of Indels^b^** **(length nt)**	**π^c ^× 10^-3^** **(SD × 10^-3^)**	**π_+_^d ^× 10^-3^** **(SD × 10^-3^)**	**π_syn _× 10^-3^**	**π_nsyn _× 10^-3^**	**Tajima's *D*-Test** **(P value)**
**PROTEIN-CODING (by gene)**								
*atpA*	501	18	0	17.30 (2.67)	---	71.08	0	0.06 (>0.1)
*atpF*	213	0	0	0	---	0	0	---
*atpI*	366	3	0	3.51 (1.43)	---	13.85	0	-1.01 (>0.1)
*cemA*	462	2	0	2.27 (4.40)	---	5.34	1.34	1.17 (>0.1)
*orf1995*	1896	8	0	1.41 (0.56)	---	2.57	1.09	-0.96 (>0.1)
*petA*	954	0	0	0	---	0	0	---
*petG*	45	0	0	0	---	0	0	---
*psaJ*	126	0	0	0	---	0	0	---
*psbA*	435	8	0	9.61 (1.46)	---	36.5	1.7	1.48 (>0.1)
*psbK*	72	0	0	0	---	0	0	---
*rpoC2*	252	6	0	13.61 (2.85)	---	10.85	14.33	2.03 (<0.05)
*rpl2*	321	2	0	3.56 (0.74)	---	7.28	2.36	1.64 (>0.1)
*rps2*	87	0	0	0	---	0	0	---
*rps3*	126	0	0	0	---	0	0	---
*rps9*	462	4	1 (21)	3.50 (1.01)	---	9.78	1.61	-0.04 (>0.1)
*rps11*	105	0	0	0	---	0	0	---
*rps12*	321	0	0	0	---	0	0	---
*rps19*	183	0	0	0	---	0	0	---
*tufA*	213	0	0	0	---	0	0	---
*ycf3*	315	2	0	1.81 (1.25)	---	3.84	1.19	-1.28 (>0.1)
*ycf4*	399	0	0	0	---	0	0	---
**INTERGENIC (by region)**								
*atpA/psbI*	343	1	0	1.75 (0.51)	1.75 (0.51)	---	---	1.22 (>0.1)
*atpF/rps11*	1556	86	16 (368)	25.61 (9.77)	30.10 (11.15)	---	---	-1.08 (>0.1)
*atpI/psaJ*	328	9	0	12.49 (2.42)	12.49 (2.42)	---	---	0.61 (>0.1)
*petG/rps3*	805	5	3 (3)	2.97 (0.44)	4.50 (0.76)	---	---	0.57 (>0.1)
*psaJ/rps12*	310	2	0	3.38 (0.65)	3.38 (0.65)	---	---	1.17 (>0.1)
*psbK/tufA*	828	4	2 (117)	1.18 (0.37)	3.47 (0.53)	---	---	0.06 (>0.1)
*psbI/cemA*	271	3	2 (2)	5.20 (1.18)	8.86 (2.00)	---	---	0.00 (>0.1)
*rpl2/rps19*	808	36	9 (164)	27.36 (5.14)	33.69 (5.70)	---	---	0.99 (>0.1)
*rps3/rpoC2*	1474	88	32 (462)	41.60 (6.65)	53.19 (9.01)	---	---	0.71 (>0.1)
*rps9/ycf4*	344	1	1 (21)	1.65 (0.53)	3.29 (0.69)	---	---	1.03 (>0.1)
*rps18/rps2-1*	1115	45	19 (418)	30.47 (9.57)	42.17 (11.33)	---	---	0.90 (>0.1)
*ycf3/ycf4*	179	0	0	0	0	---	---	---
*23S-1/23S-2*	909	6	2 (46)	3.97 (0.67)	5.28 (0.89)	---	---	2.10 (<0.05)
*23S-2/5S*	89	0	0	0	0	---	---	---

Note: *S*, number of segregating (i.e., polymorphic) sites; Indels, insertion-deletion events; π, nucleotide diversity; π_+_, nucleotide diversity including both polymorphic sites and insertion-deletion events; π_syn_, nucleotide diversity at synonymous sites; π_nsyn_, nucleotide diversity at nonsynonymous sites; SD, standard deviation.

^a ^Comprises all sites in the nucleotide alignment, including those with indels.

^b ^Indels involving more than 1 nucleotide are counted as a single event. Indel length includes the sum of all indels and includes consecutive indel events.

^c ^Only includes sites without indels.

^d ^Considers all sites, including those with indels. Consecutive indels are counted as a single polymorphic event. Indel states were measured using a multiallelic approach.

## Discussion

### Accounting for the observed values of π

At mutation-drift equilibrium, the nucleotide diversity at neutral sites should approximate 2*N*_*g*_μ [1]; thus, an essential question of this study is: are the sites that we used to measure π_silent _for the *C. reinhardtii *ptDNA neutrally evolving? We employed both noncoding sites and synonymous sites in our calculations of π_silent_; these are generally considered to be among the more neutrally evolving positions in a genome. Indeed, the nucleotide diversity at these sites within the *C. reinhardtii *ptDNA exceeds that of the more functionally constrained positions, such as first and second codon positions and rRNA-coding sites. Among the different types of silent-sites, intergenic regions have ~1.8 times more nucleotide diversity than synonymous sites. Given that synonymous sites can be subject to selection for specific tRNA anticodons, one might expect them to be under more selective constraints than intergenic regions; therefore, it is not surprising that nucleotide diversity for the intergenic regions is greater than π_syn_. Even so, because we sequenced more intergenic sites than synonymous sites, there is not a significant downward bias to our *C. reinhardtii *ptDNA-diversity measurements by including synonymous sites.

Another issue is the discrepancy in nucleotide diversity among the ptDNA loci that were studied. Factors that can result in inter-loci nucleotide-diversity discrepancy include selection (e.g., balancing-, purifying-, or positive-selection) and inconsistencies in the mutation rate across the plastid genome; however, without interspecific ptDNA-divergence data, it would be overly speculative to focus on any one of these factors. Tajima's *D*-test did yield statistically significantly positive values for two of the loci that were studied, which could be an indication of balancing selection. It is noteworthy that the magnitude of variation among the *C. reinhardtii *ptDNA loci is significantly more pronounced than what is typically observed for ptDNA: the nucleotide diversity of most plastid genomes appears to be relatively homogeneous across loci [8,13]. On the other hand, studies indicate that ptDNA substitution rates at both synonymous and intergenic sites can vary considerably among loci within a genome [14-16].

It would be ideal if we could interpret our ptDNA nucleotide-diversity measurements in relation to μ and *N*_*g*_, but this is difficult because the mutation rate for the *C. reinhardtii *plastid genome is unknown. There is evidence that μ for the mtDNA and nucDNA of *C. reinhardtii *are approximately the same [17], and consequently the disparity of π_silent _between these genomes can be explained by differences in *N*_*g *_(see [7] for a more detailed discussion). Other things being equal, in *C. reinhardtii *we would expect *N*_*g *_of the uniparentally-inherited plastid genome to be about the same as that of the mitochondrial genome, which is also uniparentally inherited, and about half that of the nuclear genome. Uniparental inheritance also implies that the organelle DNA has less opportunity for recombination during sexual reproduction compared with the nucDNA [2], meaning organelle genomes may be more prone to the influences of natural selection on linked variation (i.e., genetic hitch-hiking), which can cause *N*_*g*(organelle) _to deviate from neutral expectations (e.g., Bazin et al. [18]). Nevertheless, the only study to seriously investigate this issue with respect to the ptDNA, mtDNA, and nucDNA from a single species, *Arabidopsis lyrata*, found that *N*_*g *_of the organelle DNA and nucDNA did not depart significantly from what was expected under neutrality [8]. Thus, the fact that silent-site nucleotide diversity in *C. reinhardtii *ptDNA is only within a factor of 2 from that of the mtDNA and nucDNA can easily be accounted for by slight differences in μ and/or *N*_*g*_.

### Plastid DNA diversity for the *C. reinhardtii *ptDNA relative to that of other taxa

There is a paucity of nucleotide-diversity data from ptDNA, and the estimates that are published are limited to a small number of model land-plant species. Most of these available estimates are listed in the supplementary material of Lynch et al. [4] who compiled a summary of silent-site ptDNA diversity values from 17 land-plant species and found that on average π_silent _is 3.7 × 10^-3^, with a standard error of 1.1 × 10^-3 ^– most of these diversity data were calculated using an indels-out approach but some were generated with the indels-in method (e.g., Huang et al. [19]). More recently published π_silent _estimates from the ptDNA of land plants are concordant with these values: 0–1.2 × 10^-3 ^(*Rhododendron *spp.), ~4 × 10^-3 ^(*Machilus *spp.), and ~2 × 10^-3 ^(*Silene *spp.) [13,20,21]. In comparison, the silent-site ptDNA diversity of *C. reinhardtii *is 4 times the mean estimate for land plants (14.5 × 10^-3 ^vs. 3.7 × 10^-3^). The average π_silent _estimates from the mtDNA and nucDNA of land plants are, respectively, 0.4 × 10^-3 ^and 15.2 × 10^-3 ^[3,4]. Thus, when considering all three genetic compartments, the π_silent _values from *C. reinhardtii *match the general trend observed in land plants, with silent-site nucleotide diversity being intermediate for the plastid genome, lowest for the mitochondrial genome, and highest for the nuclear genome; however, there is an overall increase of silent-site diversity for *C. reinhardtii*, in all three of its genomes, relative to that of land plants.

To the best of our knowledge, the only species, heretofore, for which nucleotide-diversity data are available from all three genetic compartments is *A. lyrata *[8]: values of π_silent _for the ptDNA, mtDNA, and nucDNA are 1.0 × 10^-3^, 0.35 × 10^-3^, and 20 × 10^-3^, respectively. Therefore, silent-site diversity in the *A. lyrata *ptDNA is 3 times that of the mtDNA and 0.05 times that of the nucDNA. Again, the same general trend is observed for *C. reinhardtii *but with a less dramatic difference between the silent-site diversity of the organelle DNA versus that of the nucDNA.

### Addressing the mutational-hazard hypothesis

Contrary to what the mutational-hazard hypothesis forecasted, the π_silent _data for the three genetic compartments of *C. reinhardtii *do not positively correlate with genome compactness. In fact, the opposite trend is observed, with silent-site diversity being lowest for the compact mitochondrial genome (8.5 × 10^-3^), greatest for the bloated nucDNA (32.3 × 10^-3^), and intermediary for the plastid genome (14.5 × 10^-3^), which has a noncoding-DNA density that is halfway between the mtDNA and nucDNA.

Due to a lack of available data, it is difficult for us to compare π_silent _and genome-compactness values of the *C. reinhardtii *ptDNA with those of other plastid genomes; we are aware of only two ptDNA sequences for which both these data are published: those of *Arabidopsis thaliana *(π_silent(ptDNA) _= 1.4 × 10^-3^; 41% noncoding) and *Cycas taitungensis *(π_silent(ptDNA) _= 12.8 × 10^-3^; 37% noncoding) [4]. Based on their relative fractions of noncoding ptDNA, the mutational-hazard hypothesis would forecast *A. thaliana *and *C. taitungensis *to have more silent-site ptDNA diversity than *C. reinhardtii*, but instead they have less. However, it is important to stress that the π_silent _values for the *A. thaliana *and *C. tatiungensis *ptDNA are derived, in each case, from only a single locus (one protein-coding gene and one intergenic region, respectively), and, therefore, may be biased because of insufficient sampling.

If we assume that the mean π_silent _estimate of land-plant ptDNA (3.7 × 10^-3^), derived by Lynch et al. [4], is representative of the silent-site ptDNA diversity in land plants for which plastid-genome-compactness values are available (i.e., those with completely sequenced plastid genomes), then, based on the noncoding-DNA densities (Supplementary Table S1 [see Additional file 1]) the mutational-hazard hypothesis would predict less silent-site diversity for the *C. reinhardtii *ptDNA relative to the more coding-rich plastid genomes of land plants; however, *C. reinhardtii *appears to have 4 times more silent-site ptDNA diversity than the mean estimate for land plants.

Let us now compare the π_silent _and genome-compactness measurements of the *C. reinhardtii *ptDNA to those of animal mtDNA – the only organelle genomes for which these data are readily available. As highlighted earlier, the size and non-coding-DNA density of the *C. reinhardtii *plastid genome is significantly larger than that of animal mitochondrial genomes, but contrary to what would be predicted under the mutational-hazard hypothesis, the silent-site diversity of animal mtDNA is not dramatically greater than that of the *C. reinhardtii *ptDNA. Although reported π_silent _values for animal mitochondrial genomes can be as high as ~67 × 10^-3 ^(nematodes), those for arthropods (~27 × 10^-3^), birds (~17 × 10^-3^), echinoderms (~11.7 × 10^-3^), and mollusks (~13.5 × 10^-3^) are 0.8–1.9 times the π_silent _value reported here for the *C. reinhardtii *ptDNA, which is reasonably close considering the stark contrast in genome architectures.

Of the 114 kb of noncoding nucleotides in the *C. reinhardtii *plastid genome, <2 kb represent intronic DNA – the remainder are intergenic DNA. Why have intergenic nucleotides proliferated in the *C. reinhardtii *plastid genome when intronic DNA has been kept at bay? Recall, that under the mutational-hazard hypothesis the proliferation of noncoding DNA is dependent on the: 1) number of noncoding nucleotides associated with gene function (*n*); 2) per-nucleotide mutation rate (μ); and 3) effective number of genes per locus in the population (*N*_*g*_) – where the overall population-genetic barrier to noncoding-DNA colonization is defined by *N*_*g*_μ*n*. By measuring nucleotide diversity we were able to approximate 2*N*_*g*_μ; however, *n *is more difficult to estimate. For organelle introns *n *is believed to be relatively large, perhaps as high as 100 per intron [22], but *n *for organelle intergenic regions is generally unknown. One might ask, is there any reason to believe that intergenic DNA in the *C. reinhardtii *plastid genome carries a reduced burden (i.e., has fewer sites that are crucial for gene function relative to other plastid genomes)? In regards to this question, two observations are worth noting. In land plants, chloroplast genes are organized into operons, which are first transcribed into polycistronic primary transcripts and then subsequently processed into mature monocistronic units via endo- and exonucleolytic cleavage [23-25]. In *C. reinhardtii*, however, most chloroplast genes appear to be transcribed into monocistronic (or in some cases dicistronic) transcripts [26-28]. Although speculative, it is possible that the intergenic DNA in the *C. reinhardtii *plastid genome carries a reduced burden (because of a smaller *n*) relative to that of land plant ptDNA – a mutation in the intergenic DNA of land plant ptDNA could affect the expression of many genes by interfering with transcriptional or posttranscriptional steps, an outcome that seems less likely for the *C. reinhardtii *ptDNA, which has a preponderance of monocistronically expressed genes. A final comment is that in *C. reinhardtii*, genes in the mtDNA, unlike those in the ptDNA, show extensive transcriptional linkage [29] and although our estimates of 2*Ng*μ for the mitochondrial genome are low, the intergenic regions are reduced in size, which may imply that *n *for mitochondrial intergenic DNA is relatively large.

## Conclusion

The primary goal of this study was to measure nucleotide diversity for the ptDNA of *C. reinhardtii *and by doing so investigate a novel theory regarding genome evolution – the mutational-hazard hypothesis. Ultimately, the results presented in this study go against the documented trend of π_silent _positively correlating with genome compactness, and thus challenge the central premise of the mutational hazard hypothesis.

## Methods

The *C. reinhardtii *strains used in this study were obtained from the Chlamydomonas Center at Duke University. DNA was extracted from the same clonal isolate of each strain as used previously by Smith and Lee [7] for studies on the nucleotide diversity of the *C. reinhardtii *mitochondrial and nuclear genomes. PtDNA was amplified by PCR using total genomic DNA as the template; the purified PCR products were sequenced on both strands. All of the ptDNA-sequence data presented here were blasted against the *C. reinhardtii *draft nuclear genome sequence (v3.0) to insure that they are not nuclear-encoded plastid sequences (NUPTS). Our blast results suggest that very few NUPTS are in the nuclear genome (<3 kb), and the few copies that are present are highly degenerate. Nucleotide diversity and its standard deviation were calculated with DnaSP 4.5 [30]. Two different methods for calculating silent-site nucleotide diversity were employed: one that excludes indels (indels-out), which was employed for calculating π_silent_, and another that considers indels as polymorphic sites (indels-in), which was used for measuring π_silent+_. For our estimates of π_silent+_, indels involving more than one nucleotide were considered to be a single polymorphic site.

We acquired the complete plastid-genome sequence of *C. reinhardtii *strain CC-503 by assembling ptDNA sequences collected from the *C. reinhardtii *Whole Genome Shotgun Reads Trace Archive Database at GenBank. Blast hits showing >99% similarity to *C. reinhardtii *ptDNA were downloaded and assembled; all of the downloaded ptDNA sequences were subsequently blasted against the *C. reinhardtii *draft nuclear genome sequence (v3.0) to insure that no NUPTS were collected. Our assembly of the ptDNA data gave a complete CC-503 plastid genome with >50-fold coverage.

GenBank accession numbers for the ptDNA sequences produced in this study are: FJ436944–FJ436977, FJ458164–FJ458275, and FJ423446; the latter number represents the CC-503 plastid-genome sequence.

## Authors' contributions

DRS carried out the molecular studies, data analyses, and wrote the manuscript. RWL helped in interpreting the data and revising the manuscript. Both DRS and RWL have read and approved the final version of this manuscript.

## Supplementary Material

Additional file 1 **Supplementary Table S1**. The fraction of noncoding DNA in completely-sequenced plastid genomes from Streptophytes, Chlorophytes, and other plastid-harbouring taxa. Click here for file

Additional file 2 **Supplementary Table S2**. NCBI accession numbers for the plastid-DNA sequences data mined from *C. reinhardtii *strain CC-2290. Click here for file
