# Supplementary material for: Nucleotide diversity of the Chlamydomonas reinhardtii plastid genome: addressing the mutational-hazard hypothesis
Source: BMC Evol Biol. 2009 May 27;9:120. doi: 10.1186/1471-2148-9-120 (PMC2698885; doi:10.1186/1471-2148-9-120)
Supplement: Additional file 1 — Supplementary Table S1. The fraction of noncoding DNA in completely-sequenced plastid genomes from Streptophytes, Chlorophytes, and other plastid-harbouring taxa. [file 1471-2148-9-120-S1.pdf]

Supplementary Table S1 – The fraction of noncoding DNA in completely-sequenced plastid genomes.

Taxon	Genome length (nt)	% Noncoding	Genbank accession number
**PLASTID GENOMES**			
Acorus americanus	153819	42	NC_010093
Acorus calamus	153821	42	NC_007407
Adiantum capillus-veneris	150568	39	NC_004766
Aethionema cordifolium	154168	41	NC_009265
Aethionema grandiflorum	154243	41	NC_009266
Agrostis stolonifera	136584	47	NC_008591
Amborella trichopoda	162686	44	NC_005086
Aneura mirabilis	108007	46	NC_010359
Angiopteris evecta	153901	46	NC_008829
Anthoceros formosae	161162	46	NC_004543
Arabidopsis thaliana	154478	41	NC_000932
Arabis hirsuta	153689	41	NC_009268
Atropa belladonna	156687	43	NC_004561
Barbarea verna	154532	41	NC_009269
Bigelowiella natans	69166	13	NC_008408
Buxus microphylla	159010	42	NC_009599
Calycanthus floridus var. *glaucus*	153337	41	NC_004993
Capsella bursa-pastoris	154490	41	NC_009270
Carica papaya	160100	43	NC_010323
Ceratophyllum demersum	156252	42	NC_009962
Chaetosphaeridium globosum	131183	31	NC_004115
Chara vulgaris	184933	51	NC_008097
Chlamydomonas reinhardtii	204159	56	FJ423446
Chloranthus spicatus	157772	41	NC_009598
Chlorella vulgaris	150613	36	NC_001865
Chlorokybus atmophyticus	152254	38	NC_008822
Cicer arietinum	125319	39	NC_011163
Citrus sinensis	160129	43	NC_008334
Coffea arabica	155189	41	NC_008535
Crucihimalaya wallichii	155199	41	NC_009271
Cryptomeria japonica	131810	39	NC_010548
Cucumis sativus	155293	42	NC_007144
Cuscuta exaltata	125373	45	NC_009963
Cuscuta gronovii	86744	29	NC_009765
Cuscuta obtusiflora	85286	29	NC_009949
Cuscuta reflexa	121521	44	NC_009766
Cyanidioschyzon merolae strain 10D	149887	5	NC_004799
Cyanidium caldarium	164921	11	NC_001840
Cyanophora paradoxa	135599	20	NC_001675
Cycas taitungensis	163403	37	NC_009618
Daucus carota	155911	43	NC_008325
Dioscorea elephantipes	152609	41	NC_009601
Draba nemorosa	153289	41	NC_009272

Drimys granadensis	160604	43	NC_008456
Eimeria tenella strain Penn State	34750	5	NC_004823
Emiliania huxleyi	105309	13	NC_007288
Epifagus virginiana	70028	41	NC_001568
Eucalyptus globulus subsp. *globulus*	160286	43	NC_008115
Euglena gracilis	143171	52	NC_001603
Euglena longa	73345	32	NC_002652
Glycine max	152218	41	NC_007942
Gossypium barbadense	160317	43	NC_008641
Gossypium hirsutum	160301	43	NC_007944
Gracilaria tenuistipitata var. liui	183883	16	NC_006137
Guillardia theta	121524	11	NC_000926
Guizotia abyssinica	151762	41	NC_010601
Helianthus annuus	151104	41	NC_007977
Helicosporidium sp. ex *Simulium jonesii*	37454	5	NC_008100
Heterosigma akashiwo	159370	23	NC_010772
Hordeum vulgare subsp. *vulgare*	136462	47	NC_008590
Huperzia lucidula	154373	45	NC_006861
Illicium oligandrum	148553	44	NC_009600
Ipomoea purpurea	162046	40	NC_009808
Jasminum nudiflorum	165121	43	NC_008407
Lactuca sativa	152765	45	NC_007578
Lemna minor	165955	42	NC_010109
Lepidium virginicum	154743	41	NC_009273
Leptosira terrestris	195081	53	NC_009681
Liriodendron tulipifera	159886	44	NC_008326
Lobularia maritima	152659	40	NC_009274
Lolium perenne	135282	46	NC_009950
Lotus japonicus	150519	45	NC_002694
Manihot esculenta	161453	50	NC_010433
Marchantia polymorpha	121024	31	NC_001319
Medicago truncatula	124033	42	NC_003119
Mesostigma viride	118360	26	NC_002186
Morus indica	158484	43	NC_008359
Nandina domestica	156599	42	NC_008336
Nasturtium officinale	155105	41	NC_009275
Nephroselmis olivacea	200799	32	NC_000927
Nicotiana sylvestris	155941	38	NC_007500
Nicotiana tabacum	155943	38	NC_001879
Nicotiana tomentosiformis	155745	38	NC_007602
Nuphar advena	160866	44	NC_008788
Nymphaea alba	159930	43	NC_006050
Odontella sinensis	119704	16	NC_001713
Oedogonium cardiacum	196547	41	NC_011031
Oenothera argillicola	165055	44	NC_010358
Oenothera biennis	164807	44	NC_010361
Oenothera elata	165728	44	NC_002693
Oenothera glazioviana	165225	44	NC_010360
Oenothera parviflora	163365	44	NC_010362

Olimarabidopsis pumila	154737	41	NC_009267
Oltmannsiellopsis viridis	151933	42	NC_008099
Oryza nivara	134494	47	NC_005973
Oryza sativa Japonica Group	134525	42	NC_001320
Ostreococcus tauri	71666	20	NC_008289
Panax ginseng	156318	42	NC_006290
Pelargonium x *hortorum*	217942	43	NC_008454
Phaeodactylum tricornutum	117369	12	NC_008588
Phalaenopsis aphrodite	148964	46	NC_007499
Phaseolus vulgaris	150285	40	NC_009259
Physcomitrella patens subsp. patens	122890	33	NC_005087
Pinus thunbergii	119707	31	NC_001631
Piper cenocladum	160624	46	NC_008457
Platanus occidentalis	161791	44	NC_008335
Populus alba	156505	42	NC_008235
Populus trichocarpa	157033	39	NC_009143
Porphyra purpurea	191028	15	NC_000925
Porphyra yezoensis	191952	16	NC_007932
Pseudendoclonium akinetum	195867	46	NC_008114
Psilotum nudum	138829	35	NC_003386
Ranunculus macranthus	155129	42	NC_008796
Rhodomonas salina	135854	19	NC_009573
Saccharum officinarum	141182	43	NC_006084
Saccharum hybrid cultivar SP-80-3280	141182	43	NC_005878
Scenedesmus obliquus	161452	39	NC_008101
Solanum bulbocastanum	155371	41	NC_007943
Solanum lycopersicum	155461	41	NC_007898
Solanum tuberosum	155298	42	NC_008096
Sorghum bicolor	140754	49	NC_008602
Spinacia oleracea	150725	39	NC_002202
Staurastrum punctulatum	157089	43	NC_008116
Stigeoclonium helveticum	223902	51	NC_008372
Thalassiosira pseudonana	128814	15	NC_008589
Theileria parva strain Muguga	39579	17	NC_007758
Toxoplasma gondii strain RH	34996	16	NC_001799
Trachelium caeruleum	162321	48	NC_010442
Triticum aestivum	134545	46	NC_002762
Vitis vinifera	160928	44	NC_007957
Welwitschia mirabilis	119726	34	NC_010654
Zea mays	140384	43	NC_001666
Zygnema circumcarinatum	165372	45	NC_008117

Note: Complete plastid sequences were downloaded from the NCBI Reference Sequence (RefSeq) Collection on November 10, 2008; entries with incomplete Genbank annotations are not included in this table. The fraction of noncoding DNA in each genome was calculated by adding the lengths of the protein-, rRNA-, and tRNA-coding regions, subtracting this number from the total genome size, and then dividing the product of this by the full length of the genome. In all instances, intronic ORFs were considered to be noncoding DNA. Unclassified ORFs were categorized as noncoding DNA unless homologs existed in the organelle genomes of at least two other taxa.
