# Supplementary material for: Nucleotide diversity of the Chlamydomonas reinhardtii plastid genome: addressing the mutational-hazard hypothesis
Source: BMC Evol Biol. 2009 May 27;9:120. doi: 10.1186/1471-2148-9-120 (PMC2698885; doi:10.1186/1471-2148-9-120)
Supplement: Additional file 2 — Supplementary Table S2. NCBI accession numbers for the plastid-DNA sequences data mined from C. reinhardtii strain CC-2290. [file 1471-2148-9-120-S2.pdf]

Supplementary Table S2 – NCBI accession numbers for the plastid-DNA sequences data mined from *C. reinhardtii* strain CC-2290.

Accession #					
CACW10301.b1	CACW10301.g1	CACW10641.b1	CACW10641.g1	CACW11480.g1	CACW14215.b1
CACW14215.b2	CACW14215.g1	CACW14215.g2	CACW1919.b1	CACW1919.g1	CACW22128.b1
CACW23575.b1	CACW23575.g1	CACW23602.b1	CACW23882.b1	CACW24643.b1	CACW24643.g1
CACW24803.b1	CACW25840.b1	CACW25840.g1	CACW25856.b1	CACW25856.g1	CACW2634.b1
CACW2634.g1	CACW2890.b1	CACW2890.g1	CACW6507.b1	CACW6507.b2	CACW6507.g1
CACW6507.g2	CACW7106.g1	CACW7106.b1	CACW7225.b1	CACW7225.g1	CACW8237.b1
CACW8237.g1	CACW8272.b1	CACW8272.g1	CACW8423.b1	CACW8423.g1	CACW11480.b1
CACW22128.b1	CACW22128.g1	CACW23882.b1	CACW23882.g1		
